# Equitable provision of tuberculosis prevention and care for vulnerable populations

**DOI:** 10.2471/BLT.25.295448

**Published:** 2026-04-16

**Authors:** Kerri Viney, Farai Mavhunga, Annabel Baddeley, Avinash Kanchar, Tereza Kasaeva

**Affiliations:** a Department for HIV, Tuberculosis, Hepatitis and Sexually Transmitted Infections, World Health Organization, Avenue Appia 20, 1211 Geneva 27, Switzerland.

Tuberculosis affects over 10 million people each year and is a leading cause of death due to an infectious agent.^1^ Based on the latest available estimates from the World Health Organization (WHO), the disease ranks as the 10th leading cause of death worldwide.^2^ Despite being both preventable and curable, many people at high risk of developing tuberculosis do not access care, and every year, an estimated 2.4 million people with tuberculosis remain unaccounted for.^1^

Tuberculosis is transmitted through the air;^3^ therefore, anyone in close proximity to someone with untreated infectious tuberculosis is at risk of being infected. However, not everyone carries the same risk of being infected with *Mycobacterium tuberculosis* or progressing to the disease. In addition, some people may be at higher risk of poor tuberculosis treatment outcomes, while others may lack access to health care and therefore remain undiagnosed and untreated, increasing the risk of chronic illness, continued transmission and death.

**Fig. 1:**
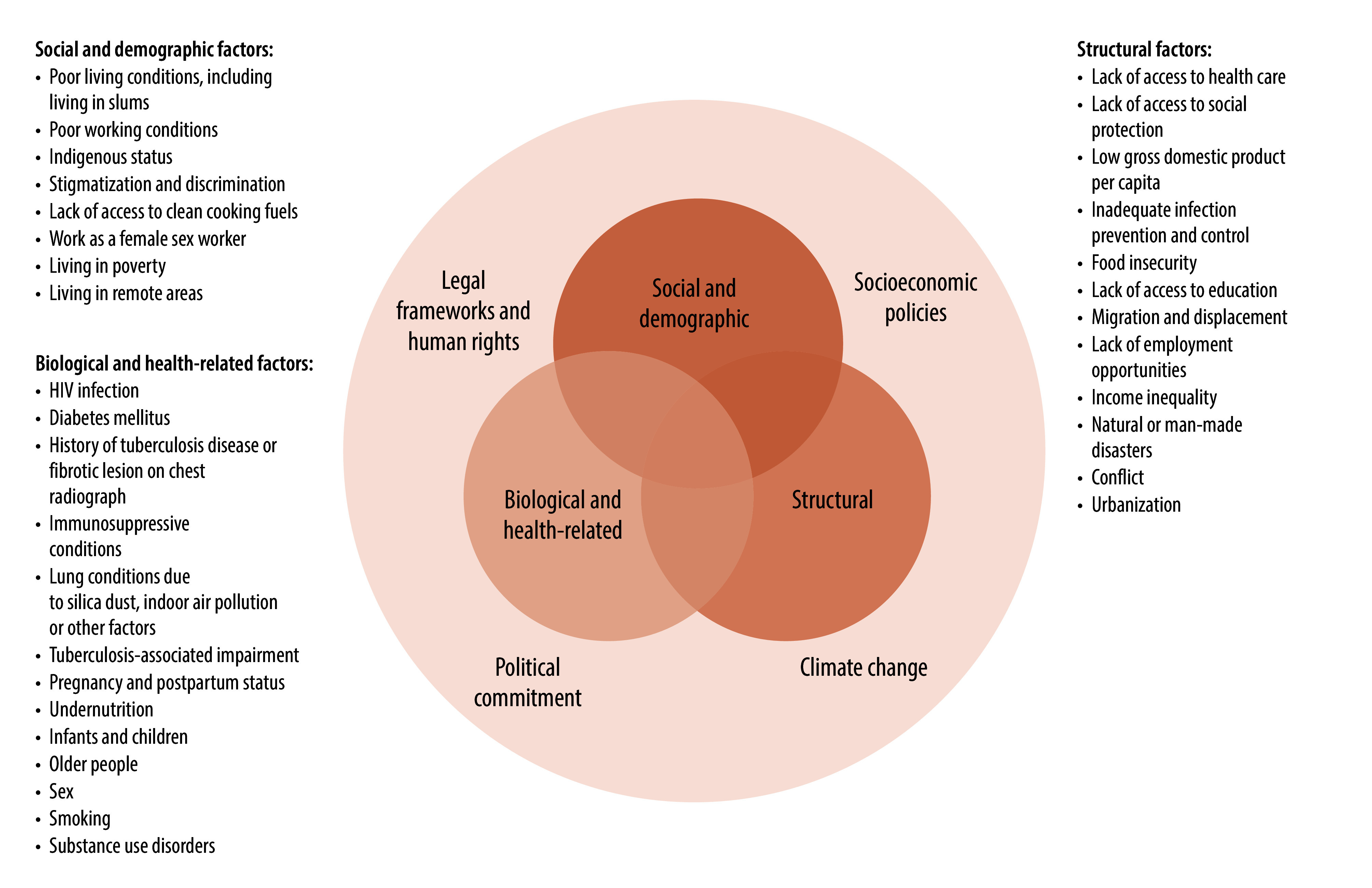
A framework describing the risk factors, drivers and determinants of tuberculosis

Globally, tuberculosis continues to be driven by upstream social determinants such as poverty, food insecurity and inadequate access to health care and social protection, as well as proximal health-related risk factors including human immunodeficiency virus (HIV) infection, smoking, diabetes, undernutrition and alcohol use disorders (Fig. 1).^4^ Systemic barriers in accessing tuberculosis services such as stigma, discrimination, legal precarity and financial hardship further compound tuberculosis-related vulnerability.

HIV: human immunodeficiency virus

Source: Adapted from WHO, 2025.^4^

Due to these determinants and risk factors, several population groups have a higher risk of developing the disease or of having poor treatment outcomes, compared with those without these risks. These groups include people in prisons; people living with HIV; people exposed to silica dust; people living in poverty, including those in urban areas; Indigenous Peoples; homeless people; migrants and refugees; people with substance use disorders; pregnant women; young children and elderly people; those with a history of tuberculosis exposure; and those living in overcrowded settlements in high-tuberculosis-burden settings. Ending the disease as a public health problem, a global goal enshrined in the WHO* End TB strategy*^5^ and the sustainable development goals,^6^ and reflected in the United Nations Political Declaration on the Fight Against Tuberculosis,^7^ will require universal access to prevention and care for all those requiring these services. As part of these efforts, attention should be paid to people who are at a higher risk of developing the disease or of having poorer outcomes.

In July 2025, WHO published a policy brief on tuberculosis among populations at high risk and for people in vulnerable situations.^4^ The policy brief presents the current scientific evidence to quantify and outline the complex reasons for the higher risk of tuberculosis faced by these populations.^8^ The brief emphasizes the importance of a mix of biomedical, social and economic interventions and policy options for an effective response to the disease, and outlines the ethical, equity and human rights imperatives for doing so.

In the policy brief, WHO has also presented a new conceptual framework for understanding tuberculosis-related vulnerability. This conceptual framework describes the biological and health-related; social and demographic; and structural risk factors, drivers and determinants of tuberculosis (Fig. 1). The framework highlights that the disease is driven by exposure to a bacterium as well as by the conditions in which people live, the health-related factors that increase risk and the structural inequities that persist in many settings. The conceptual framework also serves as a guide for action. To prevent and manage the disease effectively, the risk factors, drivers and determinants described in the conceptual framework must be systematically addressed through multisectoral action, equity-driven policies that are evidence based, and integrated health responses that leave no one behind.

Importantly, the policy brief advocates for equitable, person-centred care that upholds human rights. Achieving such care requires collaboration from all stakeholders involved in the response, including different government departments within and beyond the health sector (and beyond national borders), funders, technical partners, United Nations organizations, affected communities, civil society, nongovernmental organizations and the private sector, as well as outreach to those not yet engaged. A multisectoral response to tuberculosis that brings together these partners requires political leadership and oversight that aims to address the key determinants and drivers of the epidemic, as highlighted in *The End TB strategy* and the Multisectoral accountability framework to end TB, with important contributions from government ministries beyond health.^9^

Despite substantial evidence demonstrating that social determinants and drivers heighten vulnerability to tuberculosis, efforts to address them have remained limited for several underlying reasons, including lack of awareness, lack of political buy-in, competing priorities and chronic underfunding in several sectors, including in the health sector. As a result, multisectoral coordination may be weak or fragmented. These observations highlight an important role for high-level political and social advocacy to secure the commitments, actions and accountability mechanisms that are needed to ensure multisectoral action on tuberculosis.

Furthermore, in today’s constrained geopolitical climate where health and development funding have declined and recognizing that the tuberculosis response has been chronically underfunded for many years, the goal of strengthening multisectoral action to address the determinants and drivers of the disease may present a notable challenge. Global agencies, including WHO, have already warned of the impacts of sustained underfunding of the response, including a possible increase in tuberculosis-related deaths.^10^^,^^11^ Conversely, modelling has shown that investment in comprehensive care is cost-effective, and some tuberculosis interventions may even be cost saving.^12^

However, tuberculosis is an infectious disease born out of poverty and the disease drives poverty and social disenfranchisement. To break this deadly synergy, countries and partners must understand who is most vulnerable and at risk of tuberculosis in their settings, and this knowledge can in turn be used to target resources efficiently and to ensure the national response is sustained. In this period of diminished human and financial resources, several practical solutions can be deployed to effectively reach all people at risk of tuberculosis or with the disease. Solutions include but are not limited to priority-setting within national tuberculosis programmes so that the most impactful interventions are implemented, and further integration of services at the primary care level. Given the current reductions in available funding for tuberculosis, WHO has issued guidance on priority-setting in programme planning.^13^ The guidance aims to support countries in making informed and strategic choices about where to direct finite resources while remaining focused on equitable access to tuberculosis services, including for those most at risk.^13^

Strengthening the integration of services as part of a broader primary health-care approach can also optimize efficiencies and offers a long-term and sustainable solution to expanding equitable access to prevention and care, while ensuring that services reach those most in need, including for individuals who are most vulnerable. Decentralizing tuberculosis prevention, diagnosis and treatment to primary care level enables comprehensive care and continuity of care while improving health systems efficiencies. Services offered at the primary care level or community-based services, closer to the people and communities in need, facilitate earlier access to care and reduce barriers related to distance and cost.^14^^,^^15^ Similarly, other sectors involved in the response can also ensure the provision of services closer to where people need them (for example, addressing undernutrition, improving access to social protection or improving access to occupational screening).

Ending tuberculosis demands more than clinical solutions: it requires confronting the structural and social inequities that fuel tuberculosis-related vulnerability, as well as addressing the biological and health-related risk factors that perpetuate the disease. Ending tuberculosis also requires an understanding of who is most at risk of developing tuberculosis in a particular country, based on an understanding of the drivers and determinants of the disease in that context. This goal also requires adopting evidence-based approaches on how to best reach all people at risk, with integrated and people-centred approaches to service delivery. All stakeholders involved in the response must act with urgency and precision to identify and address vulnerability to developing the disease and to having poor tuberculosis-related outcomes. Only by doing so can we build a response that is truly inclusive, resilient and equitable, capable of ending the global tuberculosis epidemic once and for all.
